# Supplementary material for: Light-induced dimension crossover dictated by excitonic correlations
Source: Nat Commun. 2022 Feb 18;13:963. doi: 10.1038/s41467-022-28309-5 (PMC8857203; doi:10.1038/s41467-022-28309-5)
Supplement: Supplementary file 1 — Supplementary Information [file 41467_2022_28309_MOESM1_ESM.pdf]

Supplementary Information for "Light-induced dimension crossover dictated by excitonic correlations"

Yun Cheng,[1,2,*] Alfred Zong,[3,4,*] Jun Li,[5] Wei Xia,[6,7] Shaofeng Duan,[8] Wenxuan Zhao,[9] Yidian Li,[9] Fengfeng Qi,[1,2] Jun Wu,[1,2] Lingrong Zhao,[1,2] Pengfei Zhu,[1,2] Xiao Zou,[1,2] Tao Jiang,[1,2] Yanfeng Guo,[6] Lexian Yang,[9] Dong Qian,[8] Wentao Zhang,[8] Anshul Kogar,[10,†] Michael W. Zuerch,[3,4,†] Dao Xiang,[1,2,11,12,†] and Jie Zhang[1,2,11,†]

[1] *Key Laboratory for Laser Plasmas (Ministry of Education), School of Physics and Astronomy, Shanghai Jiao Tong University, Shanghai 200240, China.*
[2] *Collaborative Innovation Center of IFSA (CICIFSA), Shanghai Jiao Tong University, Shanghai 200240, China.*
[3] *Department of Chemistry, University of California at Berkeley, Berkeley, California 94720, USA.*
[4] *Materials Sciences Division, Lawrence Berkeley National Laboratory, Berkeley, California 94720, USA.*
[5] *Beijing National Laboratory for Condensed Matter Physics, Institute of Physics, Chinese Academy of Sciences, Beijing 100190, China.*
[6] *School of Physical Science and Technology, ShanghaiTech University, Shanghai 201210, China.*
[7] *ShanghaiTech Laboratory for Topological Physics, Shanghai 201210, China.*
[8] *Key Laboratory of Artificial Structures and Quantum Control (Ministry of Education), Shenyang National Laboratory for Materials Science, School of Physics and Astronomy, Shanghai Jiao Tong University, Shanghai 200240, China.*
[9] *State Key Laboratory of Low Dimensional Quantum Physics, Department of Physics, Tsinghua University, Beijing 100084, China.*
[10] *Department of Physics and Astronomy, University of California at Los Angeles, Los Angeles, California 90095, USA.*
[11] *Tsung-Dao Lee Institute, Shanghai Jiao Tong University, Shanghai 200240, China.*
[12] *Zhangjiang Institute for Advanced Study, Shanghai Jiao Tong University, Shanghai 200240, China.*

(Dated: January 26, 2022)

S1. Comparison between UED and other ultrafast probes

To unravel the dimension-dependent nonequilibrium states, it is important to identify appropriate signatures of the order parameter along different spatial axes. Though $1T$-$TiSe_2$ has been subjected to extensive studies by ultrafast probes[1–8], there lacks a technique to simultaneously characterize femtosecond evolutions of the CDW both in-plane and out-of-plane.

For instance, time-resolved terahertz or optical spectroscopy is restricted to dynamics near the Brillouin zone center[2,3,5], but the superlattice distortion and excitonic condensation take place at the Brillouin zone boundary of the non-CDW state. On the other hand, time- and angle-resolved photoemission, being a surface-sensitive probe, is able to access in-plane dynamics, but is difficult to yield information at different out-of-plane momenta at a fixed photon energy[1,4,5,8]. Femtosecond x-ray diffraction can, in principle, detect changes along different cuts of the reciprocal space. Nonetheless, the limited momentum coverage prevents a concurrent measurement of both in-plane and out-of-plane features in the same camera image[2,7], making it difficult to compare their dynamics.

Here, we use ultrafast electron diffraction (Fig. 1e), which can capture a large number of Brillouin zones with varying cross-sectional planes in a single diffraction pattern, allowing us to gain a comprehensive view of CDW dynamics along different spatial axes. In particular, anisotropic CDW fluctuations in this quasi-low-dimensional system is resolved through diffuse scatterings, whose intensity is around two orders of magnitude weaker than CDW superlattice peaks. The detection of these faint signals is made possible by the high kinetic energy of the electron pulses (3 MeV), which reduces the interfering backgrounds from multiple scattering. Using state-of-the-art pulse compression with an achromatic electron lens[9], an overall temporal resolution of 50 fs is achieved, necessary to observe the ultrafast transient phases during the structural evolution[7].

S2. Electron diffraction simulation

The CDW phase of $1T$-$TiSe_2$ is characterized by a triple-q lattice distortion that corresponds to the soft phonon at the L point in the normal state[10,11]. In terms of Miller indices, the three CDW wavevectors are

$$\mathbf{q}_1 = \left(\frac{1}{2}\ 0\ \frac{1}{2}\right), \mathbf{q}_2 = \left(0\ -\frac{1}{2}\ \frac{1}{2}\right), \\ \mathbf{q}_3 = \left(-\frac{1}{2}\ \frac{1}{2}\ \frac{1}{2}\right). \tag{S1}$$

A schematic of the periodic lattice distortion is shown in Fig. S1, where atomic displacements are in-plane and movements in adjacent $TiSe_2$ layers differ by a phase π. Mathematically, the atomic position in this modulated

* These authors contributed equally: Yun Cheng and Alfred Zong.
† Corresponding authors: A.K. (anshulkogar@physics.ucla.edu), M.W.Z. (mwz@berkeley.edu), D.X. (dxiang@sjtu.edu.cn), and J.Z. (jzhang1@sjtu.edu.cn).

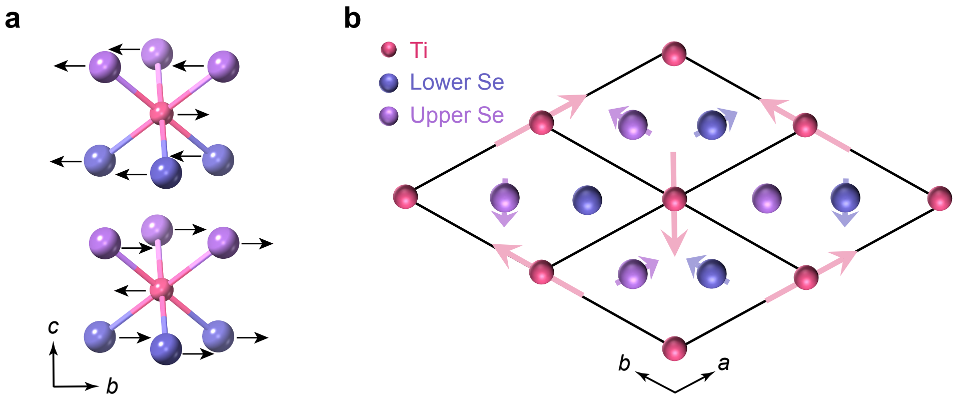

FIG. S1. **Periodic lattice distortion in the CDW phase of 1*T*-TiSe$_2$.** **a**, Schematic of atomic displacements for one particular wavevector $\mathbf{q}_1 = (\frac{1}{2}\ 0\ \frac{1}{2})$. Arrows indicate the displacement direction: In each layer, Ti and Se atoms move in-plane and in opposite directions while displacements in adjacent layers are π out of phase. **b**, Top view of the CDW in a single layer after superimposing the distortions from the triple-q wavevectors. Lengths of arrows are exaggerated.

triple-q structure is given by

$$\mathbf{R}(n,j) = \mathbf{R}_0(n,j) + \sum_{\alpha=1,2,3} \mathbf{A}_j(\mathbf{q}_\alpha)\cos\left[\mathbf{q}_\alpha \cdot \mathbf{R}_0(n,j) + \phi_j(\mathbf{q}_\alpha)\right], \quad \text{(S2)}$$

where $\mathbf{R}_0(n,j)$ is the equilibrium position of the j-th atom in the n-th unit cell without CDW modulations, and $\mathbf{A}_j(\mathbf{q}_\alpha)$ and $\phi_j(\mathbf{q}_\alpha)$ are displacement amplitude and phase. Here, $\mathbf{R}_0(n,j) \equiv \mathbf{R}_n + \mathbf{R}_j$, where $\mathbf{R}_n$ and $\mathbf{R}_j$ are positions of the n-th unit cell and the atomic position within the unit cell, respectively.

To simulate the diffraction peaks in the CDW phase, we adopt the kinematical theory of electron diffraction, which is justified by the small sample thickness (~ 60 nm) and the high electron kinetic energy (3 MeV)[12]. We denote the diffraction peak by $\mathbf{H}$, expressed as[13]

$$\mathbf{H} = h\mathbf{a}^* + k\mathbf{b}^* + l\mathbf{c}^* + m_1\mathbf{q}_1 + m_2\mathbf{q}_2 + m_3\mathbf{q}_3, \quad \text{(S3)}$$

where $(h\ k\ l)$ are the Miller indices of lattice Bragg peaks, $m_{\alpha=1,2,3}$ are integers and represent the orders of the triple-q superlattice peaks, and $\mathbf{q}_\alpha$ are CDW wavevectors given by Eq. (S1). The structure factor for diffraction from a crystal plane $\mathbf{H}$ is given by[13]

$$F_{\mathbf{H}} = \sum_{n,j} f_j \exp\left[2\pi i \mathbf{H} \cdot \mathbf{R}(n,j)\right], \quad \text{(S4)}$$

where f_j is the atomic scattering factor. Algebraic simplification transforms Eq. (S4) to[13,14]

$$F_{\mathbf{H}} = \sum_j f_j e^{2\pi i \mathbf{K}\cdot\mathbf{R}_j} \left\{ \prod_{\alpha=1}^{3} i^{-m_\alpha} e^{-i m_\alpha \phi_j(\mathbf{q}_\alpha)} J_{-m_\alpha}\left[2\pi \mathbf{H} \cdot \mathbf{A}_j(\mathbf{q}_\alpha)\right] \right\}, \quad \text{(S5)}$$

where $J_m[\cdot]$ is the Bessel function of the first kind with order m and $\mathbf{K} \equiv h\mathbf{a}^* + k\mathbf{b}^* + l\mathbf{c}^*$.

Besides the structure factor, one needs to consider an additional shape factor $S_{\mathbf{H}}$, which takes into account the fact that the Ewald sphere does not always intersect a rel-rod at its center. The shape factor for a thin sample with thickness t is[15]

$$S_{\mathbf{H}} \approx \frac{\sin(\pi\, t\, s_{\mathbf{H}})}{\pi\, t\, s_{\mathbf{H}}}, \quad \text{(S6)}$$

where $s_{\mathbf{H}}$ is the excitation error. It is defined as the reciprocal space distance between a reciprocal lattice point $\mathbf{H}$ and the Ewald sphere along the sample surface normal, given by[16]

$$s_{\mathbf{H}} \approx \frac{{K_0}^2 - |\mathbf{K_0} + \mathbf{H}|^2}{2K_0}, \quad \text{(S7)}$$

where $\mathbf{K}_0$ is the incident electron wavevector that determines the Ewald sphere. Taken together, the diffraction intensity is given by $I_{\mathbf{H}} \propto |F_{\mathbf{H}} S_{\mathbf{H}}|^2$.

For realistic diffraction intensity simulation, we also adopted a Gaussian angular distribution for the incident electron beam, whose FWHM is set to 2°. This angular spread takes into account the wrinkling nature of the suspended thin flake as well as imperfect collimation of the electron beam. The simulated electron diffraction pat-

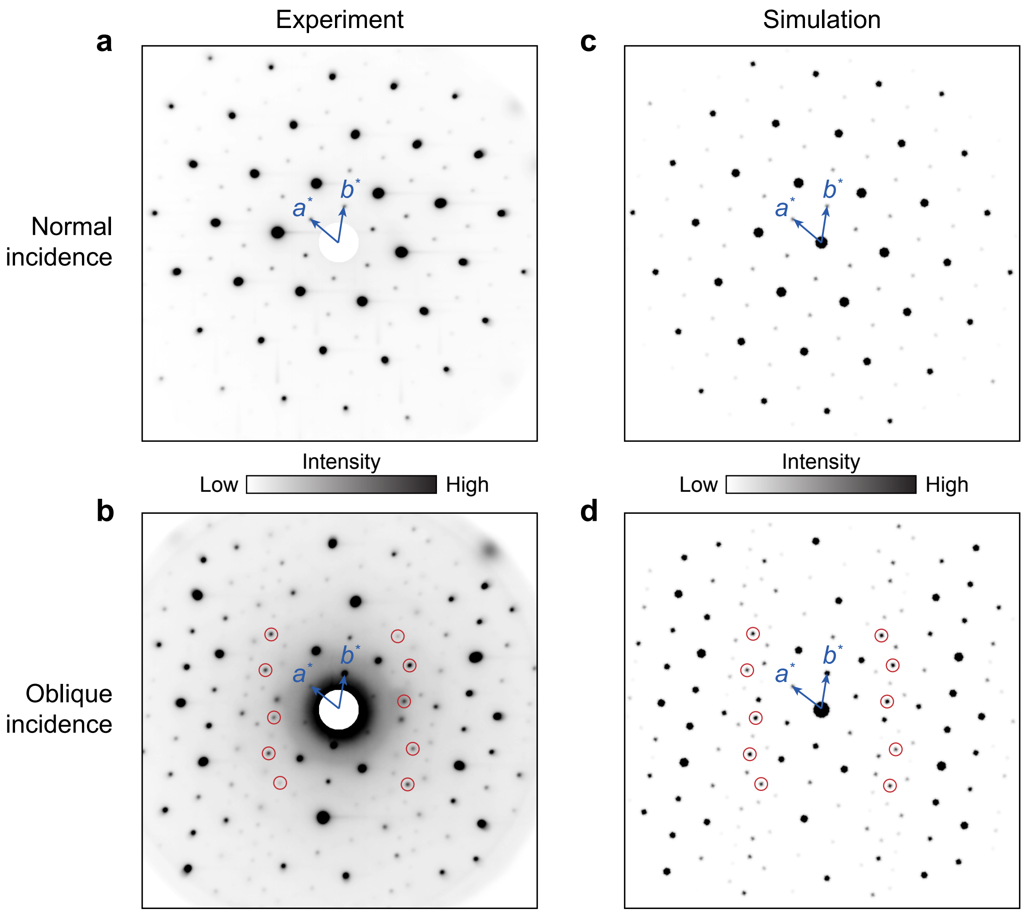

FIG. S2. **Measured and simulated electron diffraction patterns.** **a**,**b**, Experimentally measured diffraction patterns of the $2 \times 2 \times 2$ CDW phase under normal incidence (**a**) and under oblique incidence (**b**), where the electron beam is rotated from the sample surface normal by $\sim 8°$. **c**,**d**, Simulated patterns under the same conditions. Values of $\mathbf{A}_j(\mathbf{q}_\alpha)$ in Eq. (S2) are taken from ref.[10] while $\phi_j(\mathbf{q}_\alpha)$ is set to zero. Red circles mark select CDW superlattice peaks.

terns under both normal and oblique electron incidence are shown in Fig. S2, which well reproduce the experimental results.

S3. Bragg peak dynamics and the Debye-Waller effect

While the main text focuses on the CDW dynamics along the M–L cut, here we discuss the effect of photoexcitation on the primary lattice, as reflected in the lattice Bragg peaks. Figure S3a shows the intensity evolution of selected Bragg peaks up to 30 ps, where the most salient feature is a transient suppression over approximately 10 ps, which is indicative of the Debye-Waller effect due to a transient heating of the lattice. The slow timescale of the Debye-Waller effect is well separated from the fast dynamics along the M–L line, suggesting that the intensity decrease at the L point below T_c has negligible contribution from lattice heating.

Thanks to the high kinetic energy of the electron beam, we can quantitatively extract the change in the mean-squared displacement based on the Debye-Waller intensity suppression, which is dependent on the scattering wave vector $\mathbf{q}$. From the kinematical theory of electron diffraction, at each pump-probe delay t, the intensity of a Bragg peak at $\mathbf{q}$, $I(t, \mathbf{q})$, is related to the change in the mean-squared displacement, $\Delta\langle u^2\rangle(t)$, by the following equation[17]:

$$-\ln\left[\frac{I(t,\mathbf{q})}{I_0(\mathbf{q})}\right] = \frac{1}{3}\Delta\langle u^2\rangle \cdot q^2, \tag{S8}$$

where $I_0(\mathbf{q})$ is the equilibrium peak intensity. Given a collection of Bragg peaks with different q^2, we therefore expect a linear dependence of $-\ln\left[I(t)/I_0\right]$ on q^2 at each time delay t. This linear relation is confirmed by Fig. S3b, shown at a representative time step of 20 ps. We can extract $\Delta\langle u^2\rangle$ from the slope of Fig. S3b, and the evolution of $\Delta\langle u^2\rangle$ over 30 ps is shown in Fig. S3c. Consistent with the Bragg peak intensity traces, the change in the mean-squared displacement shows a characteris-

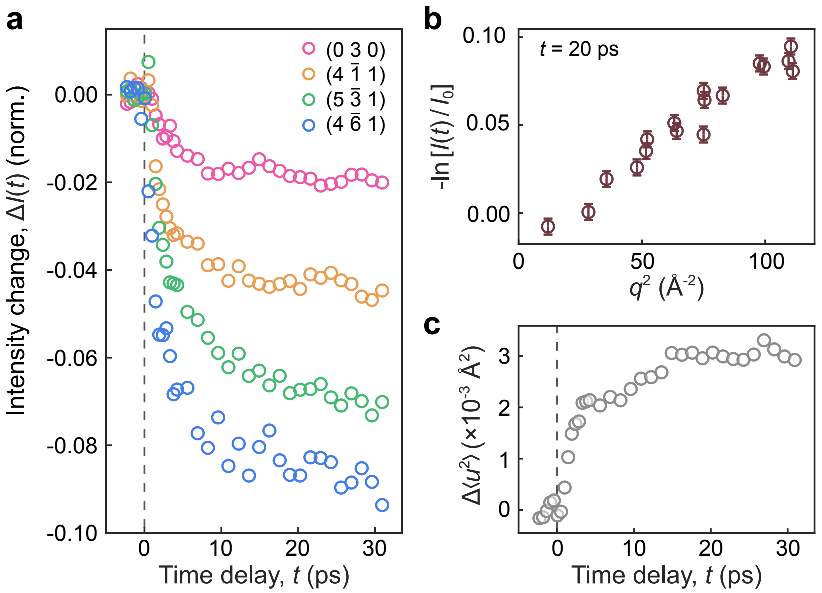

FIG. S3. **Debye-Waller effect in the Bragg peaks.** **a**, Intensity evolution of selected Bragg peaks up to 30 ps, showing suppression due to the Debye-Waller effect. **b**, Dependence of the Debye-Waller intensity drop as a function of q^2, taken at 20 ps pump-probe delay. Here, q is the magnitude of the scattering wave vector. **c**, Evolution of change in mean-squared atomic displacements, $\Delta\langle u^2\rangle$, which is derived from the slope in **b** at each delay time t (see text for details). Data in all panels were taken at 88 K with a 560 μJ/cm^2 incident fluence. Vertical dashed lines mark $t = 0$.

tic timescale over approximately 10 ps before reaching a plateau, which represents a quasi-equilibrium thermalized state.

S4. Recovery dynamics of the CDW peak

In the main text, we showed that the fluence-dependent sub-picosecond suppression of the CDW peak intensity at L remains featureless at the threshold fluence $F_c \approx 50$ μJ/cm^2 (Fig. 4d). Nonetheless, a separate fluence threshold, $\widetilde{F}_c \approx 120$ μJ/cm^2 is observed during the recovery of the CDW peak intensity at long time delay, as explained below.

Figure S4a shows several time traces of the CDW peak intensity at select incident fluences for time delays up to 30 ps. At higher pump laser fluence, the intensity recovers to a lower value as it relaxes into a quasi-equilibrium level after a few picoseconds. This quasi-equilibrium represents a metastable state that persists at least to 100 ps (Fig. S4b). We fit the long-time dynamics to a two-exponential relaxation function

$$\Delta I(t) = \frac{1}{2}\left[1+\mathrm{Erf}\left(\frac{2\sqrt{2}(t-t_0)}{w_0}\right)\right]\cdot \left(I_1 e^{-(t-t_0)/\tau_1} + I_2 e^{-(t-t_0)/\tau_2}\right), \quad \text{(S9)}$$

where w_0 characterizes the initial system response time, t_0 is determined by the pump-probe temporal overlap, $I_{1,2}$ and $\tau_{1,2}$ are the magnitude and relaxation time for the fast and slow recovery. The fitting yields $\tau_1 = 6.1 \pm 0.6$ ps and $\tau_2 = 2.5 \pm 0.6$ ns, confirming the persistent nature of the intensity plateau after the initial fast relaxation.

The fluence-dependence of the CDW peak recovery is summarized in Fig. S4d, where the transient intensities are normalized between 0 and 1 for comparison among several time delays. At delays ≥ 1 ps – the recovery portion of the dynamics – a clear kink is observed at $\widetilde{F}_c \approx 120$ μJ/cm^2, marking the onset of a steep drop of the intensity plateau at long time delay. This value corresponds to a transient complete suppression of the CDW order in parts of the sample, which is reflected in three distinct observables tracked by time-resolved photoemission experiments[5,8]: (i) upward shift of the Se-4p valence band beyond its value at T_c in equilibrium, (ii) onset of anharmonicity in the light-induced coherent A_{1g}-CDW phonon oscillation, and (iii) kink in the relaxation rate of excited electrons above the Fermi level. At fluences above $\widetilde{F}_c$, the reestablishment of the CDW order involves the formation of both in-plane and out-of-plane coherence, during which photoinduced defects can lead to a slow recovery rate and reduced diffraction intensity in the CDW peak[18,19], accounting for the rapid drop of the intensity value at long time delays in Fig. S4d.

One signature of persistent presence of photoinduced defects is observed in Fig. S4c, which shows the time evolution of in-plane width for the L-point CDW peak. The initial spike at $t < 3$ ps is associated with the melting

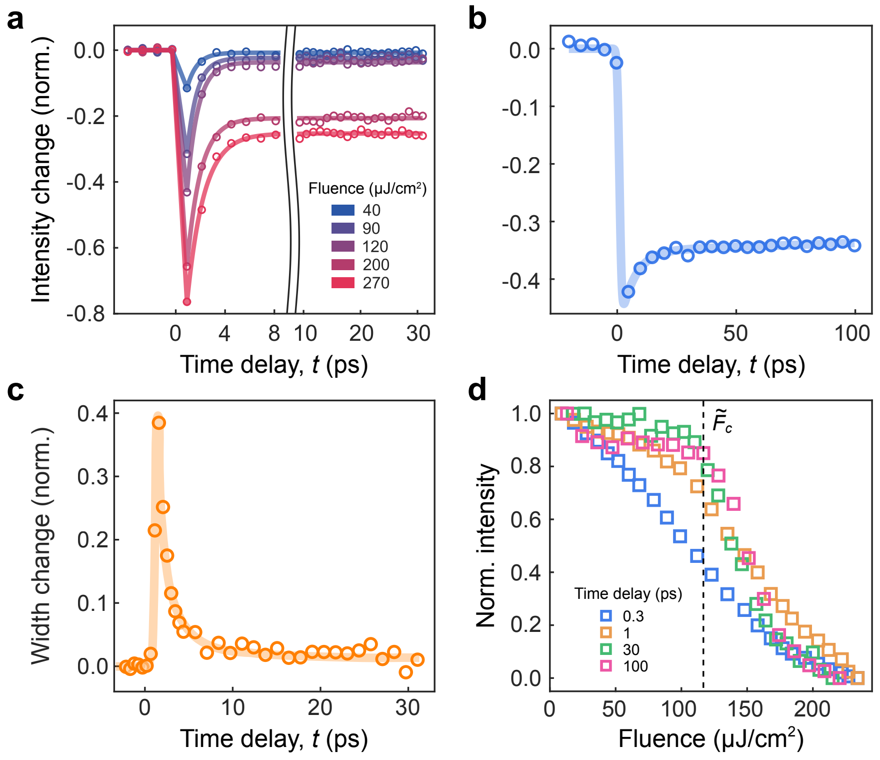

FIG. S4. **Recovery dynamics of the CDW peak.** **a**, Fluence-dependent evolutions of the CDW peak, whose intensity values are normalized by their respective pre-excitation values. Solid curves are guides to the eye. **b**, Long-time recovery dynamics of the CDW peak intensity. Blue curve is a fit to Eq. (S9). **c**, Change in the in-plane width of the CDW peak. The width, expressed in terms of FWHM, was calculated as the geometric mean between the fitted widths of 1D line profiles projected along the horizontal and vertical directions in the detector[18]. Solid curve is a guide to the eye. In **b** and **c**, data was taken with a 560 μJ/cm^2 incident pump fluence at 88 K. All values were normalized by their average prior to photoexcitation. **d**, Fluence-dependent CDW peak intensity at select time delays, where each trace is normalized between 0 and 1 for comparison. Red data points are taken from a separate sample compared to the other data points. Vertical dashed line indicates the second threshold fluence, $\widetilde{F}_c$.

of the 3D CDW order into 2D incoherent fluctuations, during which both out-of-plane and in-plane CDW coherences are significantly reduced. By 10 ps, the in-plane peak width has completed its relaxation into the quasi-equilibrium state and it stays at a plateau level that is larger than the equilibrium value. The nonzero long-time plateau in Fig. S4c suggests that the CDW correlation length is only recovered over a much longer timescale, and the slow annihilation of defects is consistent with this observation.

S5. Diffuse intensity change along the M–L cut

In Fig. 2f of the main text, we showed light-induced intensity evolutions at two selected L and M points. We affirm this result by examining momentum points in other Brillouin zones in the diffraction pattern, shown in Fig. S5. Clearly, while the CDW peak at the L point shows a sub-picosecond intensity drop with a damped coherent oscillation, diffuse intensity at other points along the M–L cut displays a transient rise, validating our interpretation of a proliferation of 2D CDW fluctuations.

To evaluate the extent of the CDW dimension crossover, we compare the absolute values of peak intensity among points along the M–L cut after photoexcitation. If one neglects the structure factor, a uniform diffuse intensity spanning across M to L indicates the maximal loss of interplane coherence. We show in Fig. S6a,b the diffraction patterns before and 1.5 ps after photoexcitation, where several peaks near the L point (labeled i, ii) and away from the L point (labeled iii, iv) are highlighted. These two images correspond to the data presented in Fig. 2e–g in the main text, taken at 88 K in the tilted geometry with a 560 μJ/cm^2 incident fluence. At $t < 0$, only sharp peaks at the L point are observed with no discernible peak away from L along the M–L line. By contrast, at $t = 1.5$ ps, points at L, M, and in between them are characterized by weak diffuse peaks of similar intensity. For a more direct comparison of absolute peak intensities along the M–L line following the 3D-to-2D dimension crossover, we plot the peak

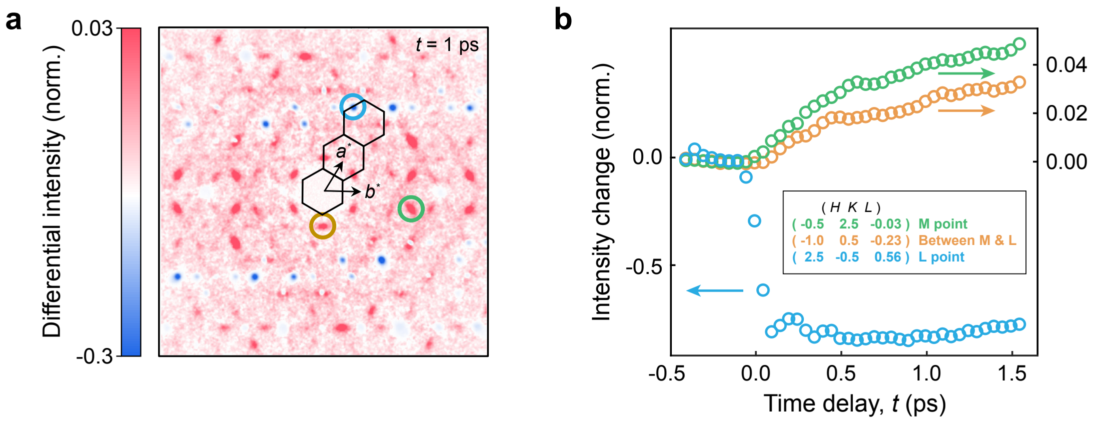

FIG. S5. **Dynamics of diffuse peaks along the M–L cut.** **a**, Differential diffraction pattern at 1 ps after photoexcitation, reproduced from Fig. 2e. Colored circles mark the peaks analyzed in **b**. **b**, Temporal evolutions of different peaks along the M–L cut. Intensities are normalized by the averaged value at $t < 0$.

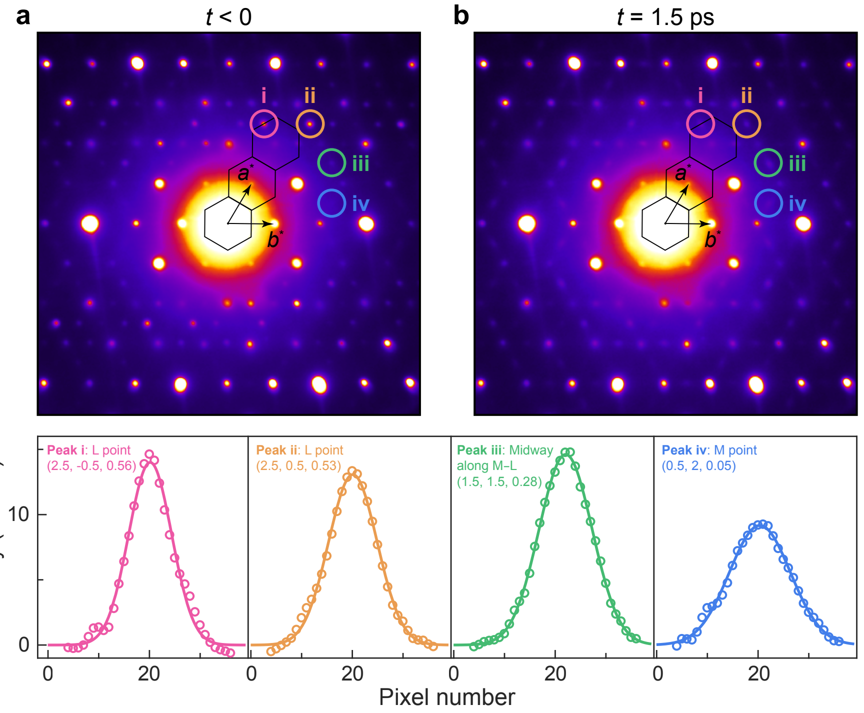

FIG. S6. **Intensity comparison between diffuse peaks along the M–L cut.** **a**,**b**, Diffraction patterns at 88 K before and 1.5 ps after the arrival of a 560 μJ/cm^2 pump pulse. Four peaks of interest along the M–L line are circled. **c**, Intensity profiles of selected peaks at $t = 1.5$ ps projected along the b^* axis, where a linear background has been subtracted. Curves are Gaussian fits.

line profiles projected along the b^* axis in Fig. S6c. The integrated intensities based on these line profiles demonstrate a comparable value along the M–L line within a factor 2, suggesting a 2D CDW state with nearly zero phase coherence between layers. The intensity variation in Fig. S6c at 1.5 ps may be attributed to the geometric structure factor, and one needs to resort to diffuse scattering simulation for a more quantitative analysis, which is beyond the scope of this paper.

S6. Distinct photoinduced changes above and below T_c

In Fig. 3 of the main text, we compared the light-induced dynamics above and below T_c at the M point. To verify that the behavior at M represents the entire M–L cut above T_c, we repeated the measurement on a separate sample with oblique electron beam incidence to access the L point in the same detector image. The differential diffraction maps in this tilted geometry below and

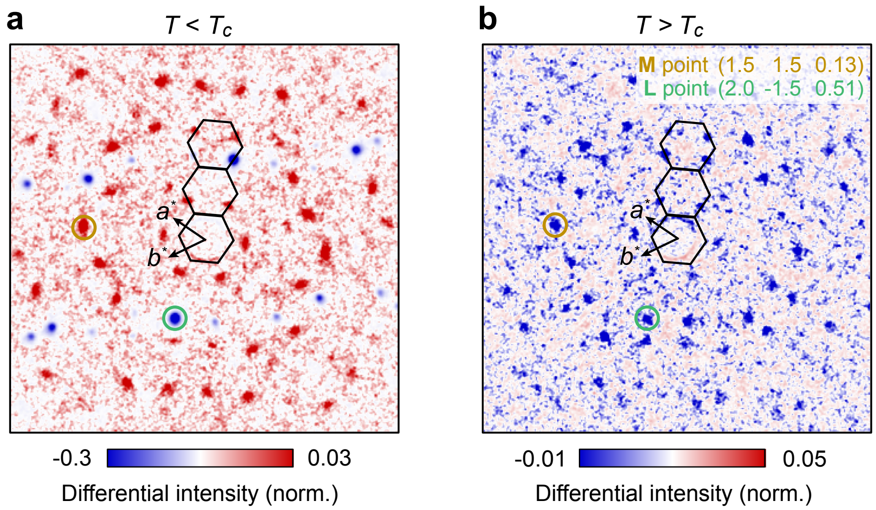

FIG. S7. **Distinct photoinduced dynamics along the M–L cut below and above T_c.** **a**,**b**, Differential diffraction pattern upon photoexciting the same sample in a tilted geometry at 88 K (**a**) and 250 K (**b**), respectively. Image was taken at 1 ps (**a**) and 0.2 ps (**b**). Yellow and green circles mark the points close to M and L respectively. The zone axis is [−9 16 82]. In both panels, intensities are normalized to respective pre-excitation values. The incident fluence was 560 μJ/cm^2.

above T_c are shown in Fig. S7, where M and L points are both visible and are marked by yellow and green circles. While the M and L points display opposite intensity changes in the 3D CDW phase below T_c (Fig. S7a), both show a transient intensity dip above T_c (Fig. S7b). The nearly identical change at M and L above T_c can be understood as a suppression of 2D CDW patches that are uncorrelated in the c-axis, which in the momentum space is represented by a diffuse rod along the M–L cut with negligible intensity modulation. In this incoherent 2D CDW state, there is hence minimal distinction between the L and M points.

Compared to the transient intensity rise at M below T_c, the light-induced loss of the 2D CDW order above T_c occurs at a much faster timescale[6], as shown in Fig. 3c in the main text. This discrepancy of timescales dictates our selection of the delay time in the differential diffraction plots in Figs. 3 and S7. It is worth noting that the fast suppression of the M point intensity above T_c (Fig. 3c) is comparable to the initial decrease of the L point intensity below T_c (Figs. 2f and S5b), which shows remarkable similarity to the ultrafast CDW dynamics in rare-earth tritellurides[20]. This similarity suggests a common origin of the two processes. Both can be interpreted as a fast decrease in the atomic displacement that is responsible for the periodic lattice distortion, and their differences mainly originate from the respective pre-excitation states: Below T_c, the displacements possess 3D long-range order; above T_c, they remain as short-range fluctuations with mostly 2D correlations.

To better visualize the two timescales of the M point dynamics below and above T_c, we show in Fig. S8 a side-by-side comparison for differential patterns at two time delays (0.2 ps and 1 ps) for two temperatures (88 K and 250 K). At 0.2 ps, we observe the opposite intensity changes at the M point by contrasting Fig. S8a and S8b. At 1 ps, the M-point suppression above T_c has partially recovered (Fig. S8d) due to the proliferation of incoherent CDW fluctuations induced by photoexcitation[21]. On the other hand, the M-point enhancement below T_c reaches its maximum around 1 ps (Fig. S8c), and the slower timescale here suggests the incoherent development of 2D CDW fluctuations.

S7. Experimental determination of the fluence threshold for excitonic melting

In the main text, we identified the fluence threshold $F_c \approx 50$ μJ/cm^2 with the critical carrier excitation density needed to completely suppress the excitonic correlations in the CDW ground state. This interpretation is drawn by comparing the fluence used in the present experiment with those reported by previous time-resolved measurements on 1T-$TiSe_2$ that employ terahertz spectroscopy[3], angle-resolved photoemission[5,8], resonant x-ray diffraction[7], and optical reflectivity[5].

For a fair comparison, we convert the *incident* fluence value $F_c \approx 50$ μJ/cm^2 to *absorbed* pump energy per normal state unit cell (u.c.) in the top-most layer of the sample, $\epsilon_{1\text{st}}$. From Beer-Lambert law, the absorbed energy density $\epsilon(z)$ follows an exponential decay

$$\epsilon(z) = \epsilon_0 e^{-z/d_\lambda}, \qquad \text{(S10)}$$

where ϵ_0 denotes the absorbed energy density on the sample surface and d_λ is the wavelength-dependent penetration depth; in our case, $d_{800\,\text{nm}} = 35$ nm[7]. From energy

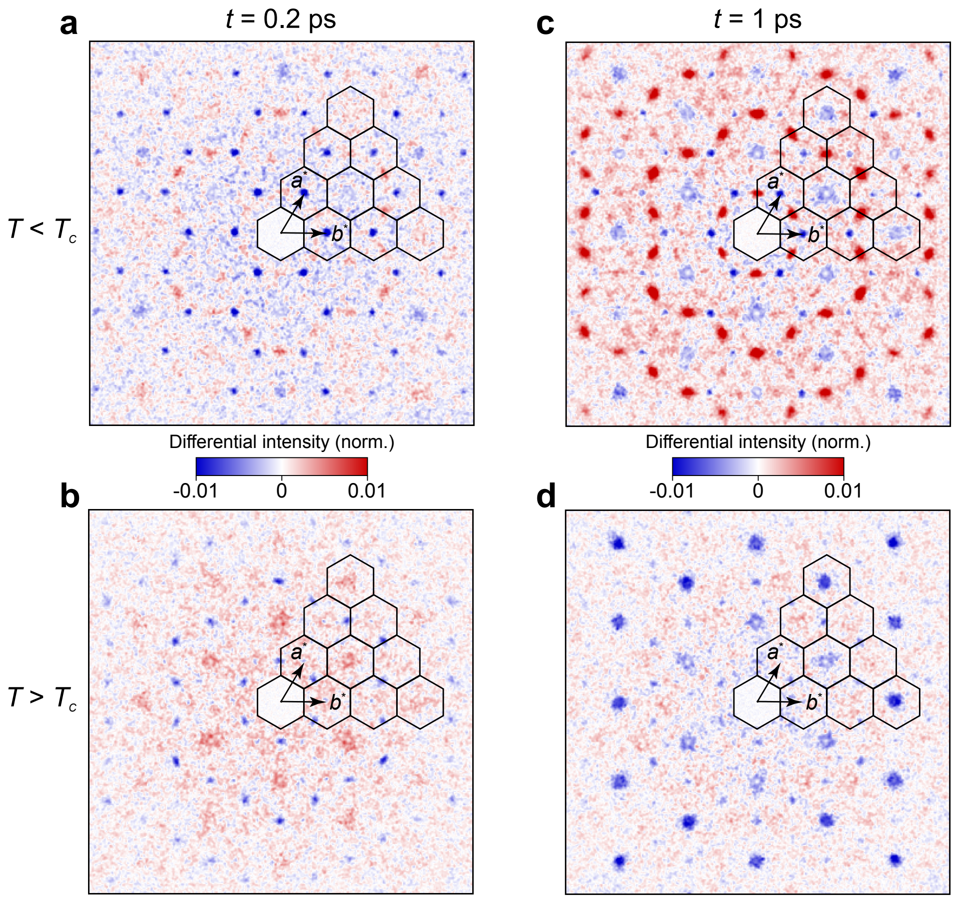

FIG. S8. **Differential patterns at normal electron incidence for selected time delays and temperatures.** Panels **a**,**c** were measured at 88 K while **b**,**d** were measured at 250 K. All panels have been symmetrized for enhanced statistics and share the same color scale, where the intensity change is normalized with respect to values prior to photoexcitation. Note that the color scale presented here is different from the one used in Fig. 3a,b in the main text. The white dots in the center of certain Bragg peaks in panels **c**,**d** are due to intensity saturation at the CCD detector. The incident fluence was 560 μJ/cm^2.

conservation,

$$F_{\mathrm{in}}(1 - R_\lambda) = \int_0^\infty \epsilon(z)\ dz = \epsilon_0 d_\lambda, \qquad \text{(S11)}$$

where F_{in} is the incident fluence and R_λ is the reflectivity at normal incidence with $R_{800\,\mathrm{nm}} = 0.68$[22]. One readily obtains

$$\epsilon_{\mathrm{1st}} = \int_0^c \epsilon(z) a^2 \sin 60^\circ\ dz, \qquad \text{(S12)}$$

where $a = 3.54$ Å and $c = 6.00$ Å are the lattice constants in the normal phase. For $F_{\mathrm{in}} = 50$ μJ/cm^2, we obtain the corresponding threshold in ϵ_{1st} as 1.8 meV/uc, which is consistent with literature values summarized in Table I in the main text. From time-resolved terahertz spectroscopy, both real and imaginary parts of the inverse dielectric function can be measured, yielding a free carrier density of 4×10^{20} cm^{-3} at this critical absorbed energy[3].

Supplementary references

[1] T. Rohwer, S. Hellmann, M. Wiesenmayer, C. Sohrt, A. Stange, B. Slomski, A. Carr, Y. Liu, L. M. Avila, M. Kalläne, S. Mathias, L. Kipp, K. Rossnagel, and M. Bauer, Collapse of long-range charge order tracked by time-resolved photoemission at high momenta, Nature **471**, 490 (2011).

[2] E. Möhr-Vorobeva, S. L. Johnson, P. Beaud, U. Staub, R. De Souza, C. Milne, G. Ingold, J. Demsar, H. Schaefer, and A. Titov, Nonthermal melting of a charge density wave in $TiSe_2$, Phys. Rev. Lett. **107**, 036403 (2011).

[3] M. Porer, U. Leierseder, J.-M. Ménard, H. Dachraoui, L. Mouchliadis, I. E. Perakis, U. Heinzmann, J. Demsar, K. Rossnagel, and R. Huber, Non-thermal separation of electronic and structural orders in a persisting charge density wave, Nat. Mater. **13**, 857 (2014).

[4] C. Monney, M. Puppin, C. W. Nicholson, M. Hoesch, R. T. Chapman, E. Springate, H. Berger, A. Magrez, C. Cacho, R. Ernstorfer, and M. Wolf, Revealing the role of electrons and phonons in the ultrafast recovery of charge density wave correlations in $1T$-$TiSe_2$, Phys. Rev. B **94**, 165165 (2016).

[5] H. Hedayat, C. J. Sayers, D. Bugini, C. Dallera, D. Wolverson, T. Batten, S. Karbassi, S. Friedemann, G. Cerullo, J. van Wezel, S. R. Clark, E. Carpene, and E. Da Como, Excitonic and lattice contributions to the charge density wave in $1T$-$TiSe_2$ revealed by a phonon bottleneck, Phys. Rev. Research **1**, 023029 (2019).

[6] M. R. Otto, J.-H. Pöhls, L. P. René de Cotret, M. J. Stern, M. Sutton, and B. J. Siwick, Mechanisms of electron-phonon coupling unraveled in momentum and time: The case of soft phonons in $TiSe_2$, Sci. Adv. **7**, eabf2810 (2021).

[7] M. Burian, M. Porer, J. R. L. Mardegan, V. Esposito, S. Parchenko, B. Burganov, N. Gurung, M. Ramakrishnan, V. Scagnoli, H. Ueda, S. Francoual, F. Fabrizi, Y. Tanaka, T. Togashi, Y. Kubota, M. Yabashi, K. Rossnagel, S. L. Johnson, and U. Staub, Structural involvement in the melting of the charge density wave in $1T$-$TiSe_2$, Phys. Rev. Research **3**, 013128 (2021).

[8] S. Duan, Y. Cheng, W. Xia, Y. Yang, C. Xu, F. Qi, C. Huang, T. Tang, Y. Guo, W. Luo, D. Qian, D. Xiang, J. Zhang, and W. Zhang, Optical manipulation of electronic dimensionality in a quantum material, Nature **595**, 239 (2021).

[9] F. Qi, Z. Ma, L. Zhao, Y. Cheng, W. Jiang, C. Lu, T. Jiang, D. Qian, Z. Wang, W. Zhang, P. Zhu, X. Zou, W. Wan, D. Xiang, and J. Zhang, Breaking 50 femtosecond resolution barrier in MeV ultrafast electron diffraction with a double bend achromat compressor, Phys. Rev. Lett. **124**, 134803 (2020).

[10] F. J. Di Salvo, D. E. Moncton, and J. V. Waszczak, Electronic properties and superlattice formation in the semimetal $TiSe_2$, Phys. Rev. B **14**, 4321 (1976).

[11] M. Holt, P. Zschack, H. Hong, M. Y. Chou, and T.-C. Chiang, X-ray studies of phonon softening in $TiSe_2$, Phys. Rev. Lett. **86**, 3799 (2001).

[12] P. Zhu, Y. Zhu, Y. Hidaka, L. Wu, J. Cao, H. Berger, J. Geck, R. Kraus, S. Pjerov, Y. Shen, R. I. Tobey, J. P. Hill, and X. J. Wang, Femtosecond time-resolved MeV electron diffraction, New J. Phys. **17**, 063004 (2015).

[13] T. Janssen, A. Janner, A. Looijenga-Vos, and P. M. de Wolff, Incommensurate and commensurate modulated structures, in *International Tables for Crystallography*, Vol. C, edited by E. Prince (Springer Netherlands, Dordrecht, 2006) pp. 907–955.

[14] J. Li, J. Li, K. Sun, L. Wu, H. Huang, R. Li, J. Yang, X. Shen, X. Wang, H. Luo, R. J. Cava, I. K. Robinson, Y. Zhu, W. Yin, and J. Tao, Ultrafast decoupling of atomic sublattices in a charge-density-wave material (2019), arXiv:1903.09911.

[15] B. Fultz and J. M. Howe, Diffraction from crystals, in *Transmission Electron Microscopy and Diffractometry of Materials* (Springer, Berlin, Heidelberg, 2002) pp. 225–274.

[16] J. M. Zuo and J. C. Spence, *Advanced Transmission Electron Microscopy* (Springer, New York, 2017).

[17] K. Sokolowski-Tinten, X. Shen, Q. Zheng, T. Chase, R. Coffee, M. Jerman, R. K. Li, M. Ligges, I. Makasyuk, M. Mo, A. H. Reid, B. Rethfeld, T. Vecchione, S. P. Weathersby, H. A. Dürr, and X. J. Wang, Electron-lattice energy relaxation in laser-excited thin-film Au-insulator heterostructures studied by ultrafast MeV electron diffraction, Struct. Dynam. **4**, 054501 (2017).

[18] S. Vogelgesang, G. Storeck, J. G. Horstmann, T. Diekmann, M. Sivis, S. Schramm, K. Rossnagel, S. Schäfer, and C. Ropers, Phase ordering of charge density waves traced by ultrafast low-energy electron diffraction, Nat. Phys. **14**, 184 (2018).

[19] A. Zong, A. Kogar, Y.-Q. Bie, T. Rohwer, C. Lee, E. Baldini, E. Ergeçen, M. B. Yilmaz, B. Freelon, E. J. Sie, H. Zhou, J. Straquadine, P. Walmsley, P. E. Dolgirev, A. V. Rozhkov, I. R. Fisher, P. Jarillo-Herrero, B. V. Fine, and N. Gedik, Evidence for topological defects in a photoinduced phase transition, Nat. Phys. **15**, 27 (2019).

[20] A. Zong, P. E. Dolgirev, A. Kogar, Y. Su, X. Shen, J. A. W. Straquadine, X. Wang, D. Luo, M. E. Kozina, A. H. Reid, R. Li, J. Yang, S. P. Weathersby, S. Park, E. J. Sie, P. Jarillo-Herrero, I. R. Fisher, X. Wang, E. Demler, and N. Gedik, Role of equilibrium fluctuations in light-induced order, Phys. Rev. Lett. **127**, 227401 (2021).

[21] P. E. Dolgirev, M. H. Michael, A. Zong, N. Gedik, and E. Demler, Self-similar dynamics of order parameter fluctuations in pump-probe experiments, Phys. Rev. B **101**, 174306 (2020).

[22] S. C. Bayliss and W. Y. Liang, Reflectivity, joint density of states and band structure of group IVb transition-metal dichalcogenides, J. Phys. C: Solid State Phys. **18**, 183327 (1985).
